# Exploring the Relationship Between Dietary Habits and Perceptions of Mental and Physical Disorders, or a Sense of Accomplishment in Japan

**DOI:** 10.3390/nu16213702

**Published:** 2024-10-30

**Authors:** Tsuyoshi Iwasa, Kouji Satoh, Makoto Hazama, Hiroyo Kagami-Katsuyama, Naohito Ito, Mari Maeda-Yamamoto, Jun Nishihira

**Affiliations:** 1 Health Information Science Center, Hokkaido Information University, Ebetsu 069-0832, Japan; iwasa@mec.edc.ac.jp (T.I.); ks46317@do-johodai.ac.jp (K.S.); m-hazama@do-johodai.ac.jp (M.H.); k.katsuyama@do-johodai.ac.jp (H.K.-K.); n_ito@do-johodai.ac.jp (N.I.); 2 Media Education Center, Electronics Development Computer College, Ebetsu 069-0832, Japan; 3 Institute of Food Research, National Agriculture and Food Research Organization, Tsukuba 305-8642, Japan; marimy@affrc.go.jp

**Keywords:** dietary habits, mental and physical stress, psychosomatic disorders, sense of achievement

## Abstract

Background. Japanese dietary patterns have traditionally focused on vegetables, legumes, and fish; however, in the last few quarters of the century, the consumption of meat, processed food, and ultra-processed food has become popular. It is anticipated that these changes in the Japanese dietary environment will increase the risk of developing psychosomatic disorders. Methods. In this study, we examined the relationship between dietary habits, psychosomatic disorders, and a sense of achievement. For men and women aged 20–80 years (*n* = 851) living in and around Ebetsu City, Hokkaido, Japan, a questionnaire on dietary habits over the past year, including mental and physical stress and a sense of achievement, was administered. The associations between dietary habits and psychosomatic disorders or a sense of achievement were analyzed using logistic regression (*n* = 654). Results. The associations between dietary habits and psychosomatic disorders differ depending on sex and age. We found that subjects who consumed more processed meat and ultra-processed food reported more psychosomatic disorders. On the other hand, those who perceived a sense of achievement consumed higher amounts of vegetables, albeit with a lower fish intake. Conclusions. In this study, we demonstrated that dietary habits and food ingredients are associated with mental and physical stress and a sense of achievement. Fish, which is considered to be good for the body, was mostly consumed by subjects who experienced mental and physical stress. These data indicate that highly stressful conditions may encourage fish consumption, as the body needs to mitigate psychosomatic disorders.

## 1. Introduction

Psychosomatic disorders include psychophysiological disorders, somatic symptom disorders, and the broader category of somatic symptom and related disorders. The evolution of these terms reflects a growing understanding of the complex interplay between the mind and body in health and disease [1,2,3,4]. Stress affects our bodies in many ways and can affect several physical and mental conditions. Stress management can go a long way in helping to manage psychosomatic disorders. Modern society is considered to be stressful. Stress can cause various diseases, particularly mental disorders. Mental disorders, including anxiety disorders and depression, affect one in eight people worldwide [5]. Various mental illnesses caused by stress have a greater impact on social and economic activities, with economic losses exceeding health care costs [6,7,8]. Additionally, long-term illness has become a significant burden, not only for the individual but also for their family. In Japan, approximately 1.2 million patients with mental illness were reported in 2020 [9], and stress countermeasures have become an important problem. According to “Healthy Japan 21” [10], in order to maintain the mental health, a balanced diet is one of the basic elements for managing stress, which is indispensable for mental well-being.

Stress reactions can be acute or chronic. Noradrenaline is secreted in response to acute stress and activates the sympathetic nervous system when exposed to stressors. In addition, adrenaline secretion amplifies the activity of the sympathetic nervous system. This leads to physical reactions, such as increased blood pressure and sweating, and mental reactions, such as anger and fear. When exposed to stress, the consumption of high-energy diets (sweet and oily) is promoted as a coping mechanism [11,12,13]. In the case of acute stress, one can recover physical and mental states by resolving stress.

However, prolonged exposure to stressors can also lead to chronic stress. Chronic stress conditions can cause feelings of sadness, anxiety, and depression, and physiological exhaustion can lead to fatigue and an increased risk of illness [14]. During chronic stress, the stress hormone cortisol is constantly secreted. This results in increased appetite and an inability to restrict food consumption, which can lead to the development of lifestyle-related diseases [15]. As such, stress increases the risk of disease development, and appropriate stress control is important for health maintenance.

Healthy Japan 21 suggests well-balanced nutrition and dietary habits as one of the measures to counter stress. In acute-stress conditions, not only high-energy foods but also proteins and vitamin C are required [11,16,17,18] to respond to the physical responses. In addition, there has been much research on dietary patterns that influence the risk of developing mental disorders. Furthermore, recent studies have shown that a diet rich in whole foods such as fruits, vegetables, and whole grains may lower the risk of developing mental health conditions [19,20]. Additionally, consuming a balanced diet with adequate amounts of omega-3 fatty acids and other essential nutrients can have a positive impact on mental health [21].

The Mediterranean dietary pattern is characterized by a predominance of vegetables; consumption of fruits and nuts, olive oil, fish, and dairy products; and low consumption of red and processed meat. The DASH diet is similar to the Mediterranean diet but with restricted consumption of fat-free or low-fat dairy products, saturated fatty acids, and sugar [22]. The Mediterranean and DASH diets have been reported to reduce the risk of developing depression and anxiety [22]. In a further study on food frequency and the occurrence of depression and anxiety in the developing world, it was reported that individuals with high fruit consumption are at a lower risk of mental breakdown [23]. In contrast, Western diet patterns are characterized by high consumption of red and processed meat, refined cereals, sweet beverages, high-fat foods, butter, and potatoes, and low consumption of vegetables and fruits. Individuals with Western dietary patterns have been reported to be at an increased risk of developing anxiety and depression [24]. More recently, the relationship between processed foods and mental disorders has been investigated, and it has been reported that higher consumption of processed foods is associated with the onset of a higher risk [25]. Dietary patterns and certain foods are known to influence the risk of developing mental health disorders.

The Japanese diet has traditionally been a dietary pattern centered on vegetables and fish. Japanese dietary patterns and health conditions have been studied, and it has been reported that Japanese dietary patterns around 1975 inhibited lipid accumulation, thereby preventing obesity and aging [26]. The essential cause is not the PFC balance of the diet; instead, the type and quantity of nutrients obtained by the diet are more important. In addition, the rice-based Japanese diet was beneficial not only for physical health but also for mental health when the consumption of the other foods was correlated with the consumption of rice (e.g., miso sap and natto), and the correlated foods were the central ingredients in the Japanese dietary pattern around 1975 [26]. However, in the past few decades, owing to lifestyle changes, the consumption of rice has decreased, and the consumption of refined cereals and animal products such as bread, processed meat, and dairy products has increased [27]. In addition, many processed foods (processed food and ultra-processed food) are popular and incorporated into everyday life. It is anticipated that such a dietary pattern in Japan in recent decades will increase the risk of stress and mental disorders [28].

The aim of this article is to explore the relationship between dietary habits and psychosomatic disorders or a sense of achievement among Japanese males and females living in a local area. The uniqueness of this study is two-fold. First, in analyzing the relationship between dietary habits and physical/mental well-being, we utilized a survey on dietary habits from the previous year using the FFQ (Food Frequency Questionnaire) for individuals in a city and its surrounding area in Japan. Because the FFQ covers a broad range of dimensions in dietary habits, we can delve into more details on food consumption. Second, we used two questionnaire items developed as indicators of long-term stress. These questions addressed negative (psychosomatic disorders) and positive (a sense of achievement) subjective perceptions of individual life over the past year. With this setup, we analyzed the relationship between stress conditions and dietary habits over one year, searched for foods related to stress, and examined the relationship between stress and food ingredients in the pattern of the Japanese diet.

## 2. Materials and Methods

### 2.1. Study Design

In this study, we conducted the Sukoyaka Healthy Survey for individuals including adult males and females (aged 20–80 years) living in Ebetsu City, Hokkaido, and its suburbs, in fiscal years 2019 and 2020 [29]. Subjects who provided informed consent were surveyed twice a year for background information, physical information, and various questionnaires (sleep, stress, diet, etc.). This study was approved by the Bioethics Committee of Hokkaido Information University (approval date: 22 April 2019; approval number: 2019-04).

### 2.2. Measurement of Stress Conditions, Dietary Habits, and Confounders

The present analysis used unique questions to investigate the stress status in the past year. Questions regarding the “actual feeling of mental and physical stress in the past year” and the “actual feeling of achievement in the past year” were evaluated on an 11-point scale from 0 to 10 (0 indicating no feeling at all, and 10 indicating an intense feeling never experienced before). We condensed data on these 11-point scales into dummy variables, which indicate whether the scale is greater than or equal to the median value for the subsample. We defined these as $1\left[“actual\;feeling\;of\;mental\;and\;physical\;stress”\geq median\right]$ for psychosomatic disorders and $1\left[“actual\;feeling\;of\;achievement”<median\right]$ for a sense of achievement.

A food frequency questionnaire (FFQ) was used to assess dietary habits over the past year, and food consumption and habits were investigated. The original description for the FFQ is in [30]. One survey in the summer and one in the winter were conducted on the FFQ, and one survey on mental and physical weakness and a sense of achievement over the last year was conducted.

In the analysis described below, we also use data on background factors such as body mass index (BMI), systolic blood pressure (SBP), housemates, educational background, employment status, and cohabitants for individuals. Using data on housemates, we construct three dummy variables indicating whether the participants were living with (1) a spouse, (2) any child, or (3) any parent. With educational background data, we construct indicator variables for “some college education or associate degree” (School (junior college)) and “bachelor’s degree or higher” (School (university)). We also included in the confounders for our analysis a dummy variable indicating that the individual works full-time.

### 2.3. Data Collection

**Figure 1:**
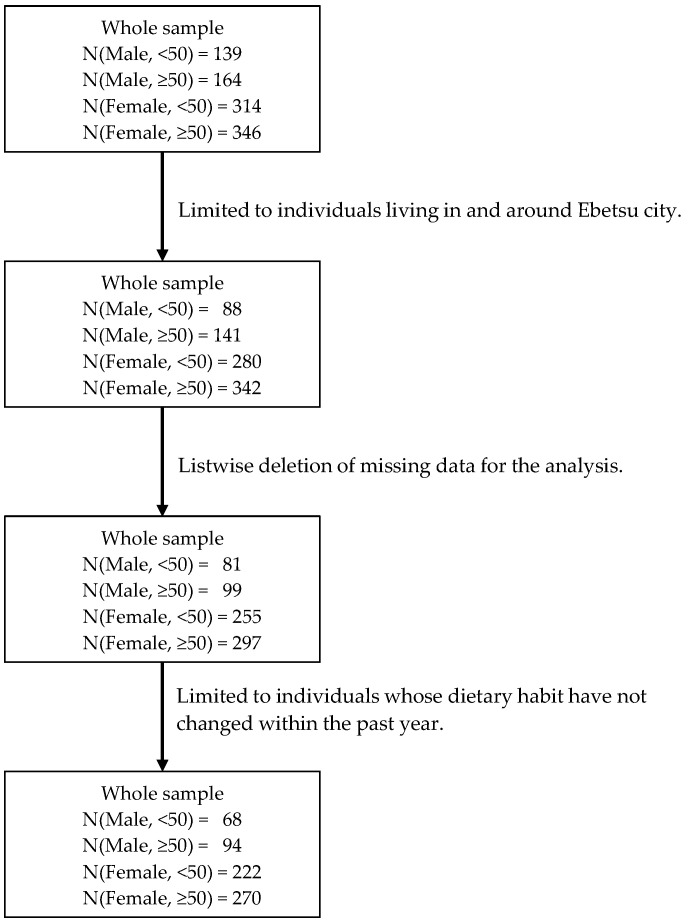
Data selection process.

Figure 1 shows our data selection process. While the survey covers areas beyond Ebetsu city and its surroundings, we limited the analysis to the Ebetsu area due to data integrity based on a sufficient subsample size (*n* = 851). When we limited our sample by listwise deletion of missing data for our analysis, the sample size shrank to *n* = 732. Finally, we excluded individuals from our dataset who had changed their dietary habits within the past year (*n* = 654), because physical and mental disorders, as well as a sense of achievement, were measured over the past year.

### 2.4. Statistical Analysis

First, we summarized the descriptive statistics of our sample. Then, we investigated the association between dietary habits and psychosomatic disorders, or a sense of achievement, while controlling for certain confounders.

To explore the relationships between dietary habits and psychosomatic disorders or a sense of achievement, we conducted logistic regression analyses. We divided the sample into four groups: (1) males under 50 years old, (2) males aged 50 or older, (3) females under 50 years old, and (4) females aged 50 or older. Our two dependent variables are follows: (1) an indicator of whether the respondent experienced psychosomatic disorders at or above the median level in the subsample, and (2) an indicator of whether the respondent experienced a sense of achievement below the median level in the subsample. The measure for psychosomatic disorders is an eleven-point Likert scale response to the question “To what extent have you experienced physical and/or mental disorders over the past year?”. The sense of achievement is assessed using an eleven-point Likert scale response to the question “To what extent have you achieved what you wanted to do or what you had to do over the past year?”. Supplementary Table S3 provides the results of validation analysis for these two variables as measures of individual stress condition. We performed regressions for each FFQ item. In every regression, we included BMI, SBP, dummy variables for housemates, educational background, and an indicator of full-time employment as confounders. We report odds and their 95% confidence intervals for every regression.

## 3. Results

The summary statistics by subsamples are shown in Table 1. For both genders, the mean SBP in younger individuals is significantly lower than that in older individuals (*p* < 0.0001). The means of psychosomatic disorders and a sense of achievement do not differ between age groups within each gender at the 5% significance level.

**Figure 2:**
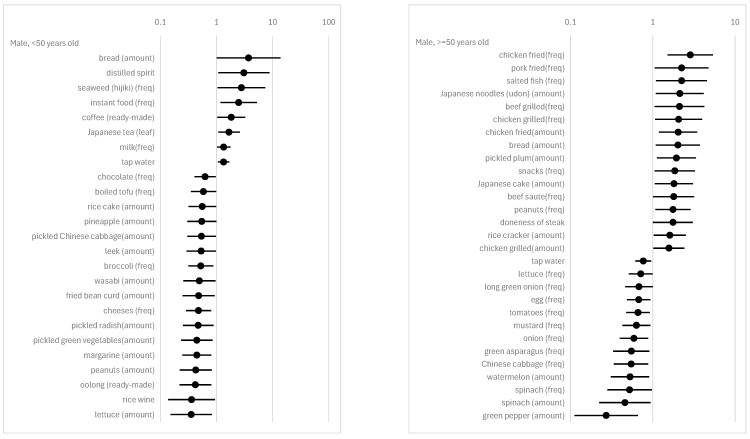

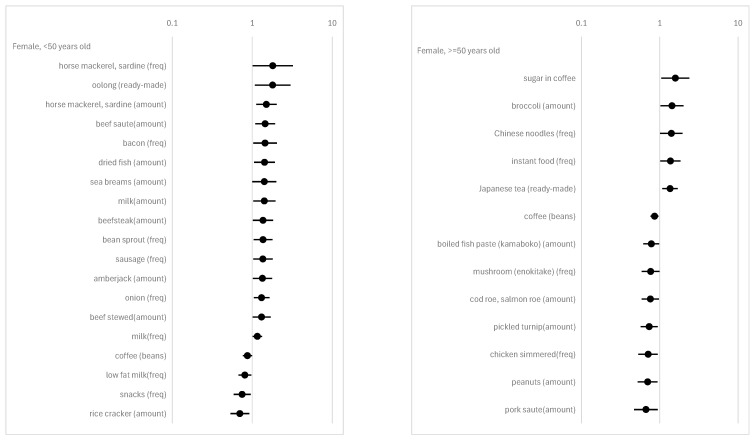
Odds of FFQ items on psychosomatic disorders. Notes: This graph shows the odds for items with 5% significance from the FFQ. Black circle markers represent point estimates, and black solid lines depict 95% confidence intervals. The dependent variable of logistic regression is 1 [“actual feeling of mental and physical stress” ≥ median].

**Figure 3:**
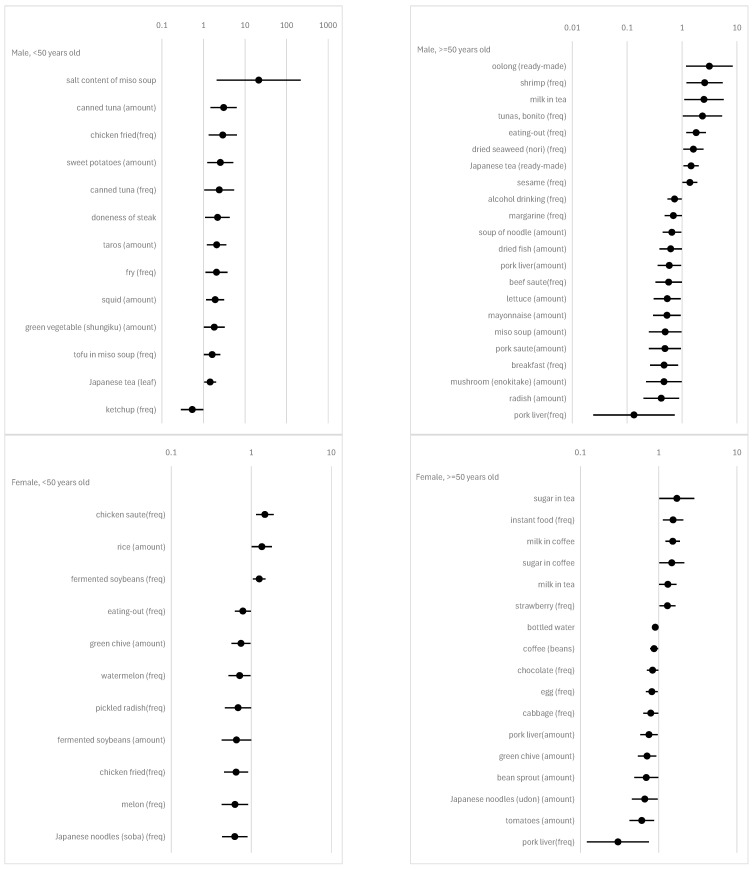
Odds of FFQ items on a sense of achievement. Notes: This graph shows the odds for items with 5% significance from the FFQ. Black circle markers represent point estimates, and black solid lines depict 95% confidence intervals. The dependent variable of logistic regression is 1 [“actual feeling of achievement” < median].

We marshaled all significant (α = 0.05) results of the subsample in Figure 2 and Figure 3. The complete results, including insignificant estimates, are provided in Supplementary Figures S1 and S2. Note that the correlations between dietary habits and psychosomatic disorders or a sense of achievement mentioned below are all adjusted for confounders. When we see the results of the regression analysis, it is also important to consider that the dependent variables are defined as $1\left[“actual\;feeling\;of\;mental\;and\;physical\;stress”\geq median\right]$ for psychosomatic disorders and $1\left[“actual\;feeling\;of\;achievement”<median\right]$ for a sense of achievement. The results vary across genders and age groups.

With regards to alcohol, the frequency of alcohol drinking is positively associated with a sense of achievement in males aged 50 and older (Figure 3, top right). The amounts of rice wine and distilled spirits consumed per occasion show a negative and positive correlation, respectively, with psychosomatic disorders in males under 50 years old (Figure 2, top left).

In the category of rice and miso soup, which are traditional Japanese principal foods, the amount of rice consumed per occasion is negatively correlated with a sense of achievement in females under 50 years old (Figure 3, bottom left). The amount of miso soup consumed per occasion is positively correlated with a sense of achievement in males aged 50 and older (Figure 3, top right). The salt content in miso soup is negatively correlated with a sense of achievement in males under 50 years old (Figure 3, top left). Note that the salt content in miso soup as an FFQ item does not ask the respondent about the amount of salt added to the miso soup, but rather the level of saltiness in the miso soup. The question for this item is “How is the miso soup seasoned?”. The responses are 0 = mild, 1 = normal, and 2 = strong.

Regarding meat consumption, the results show a marked difference between subsamples. For males under 50 years old, among meat items, only the frequency of fried chicken consumption negatively correlates with a sense of achievement (Figure 3, top left). In contrast, for males aged 50 and older, many more meat items show a positive correlation with psychosomatic disorders or a sense of achievement: seven items (chicken fried (freq), pork fried (freq), beef grilled (freq), chicken grilled (freq), chicken fried (amount), beef, sauteed (freq), and chicken grilled (amount)) for psychosomatic disorders and four items (pork liver (freq), pork, sauteed (amount), beef, sauteed (freq), and pork liver (amount)) for a sense of achievement (Figure 2 and Figure 3, top right). For females under 50 years old, five items (beef, sauteed (amount), bacon (freq), beefsteak (amount), sausage (freq), and beef stewed (amount)), which are all different from those for males aged 50 and older, are positively correlated with psychosomatic disorders (Figure 2, left). Additionally, one item (chicken, sauteed (freq)) is negatively correlated and another item (chicken, fried (freq)) is positively correlated with a sense of achievement (Figure 3, bottom left). For females aged 50 and older, two items (pork, sauteed (amount), and chicken, simmered (freq)) are negatively correlated with psychosomatic disorders (Figure 2, bottom right), and two other items (pork liver (freq) and pork liver (amount)) are positively correlated with a sense of achievement (Figure 3, bottom right).

Dairy items are associated only with psychosomatic disorders, and only for individuals under 50 years old. The frequency of milk consumption is positively correlated with psychosomatic disorders in males and females under 50 years old (Figure 2, left). The amount of milk consumed per occasion is positively correlated with psychosomatic disorders in females under 50 years old (Figure 2, bottom left). The frequency of low-fat milk consumption is negatively correlated with psychosomatic disorders in female under 50 years old (Figure 2, bottom left). The frequency of cheese consumption is negatively correlated with psychosomatic disorders in males under 50 years old (Figure 2, top left).

The frequency of egg consumption is negatively correlated with psychosomatic disorders in males aged 50 and older (Figure 2, top right). Conversely, it is positively correlated with a sense of achievement in females aged 50 and older (Figure 3, bottom right).

As for fish and seafood, it is notable that five items (horse mackerel, sardine (freq), horse mackerel, sardine (amount), dried fish (amount), sea breams (amount), and amberjack (amount)) are positively correlated with psychosomatic disorders, and no item is associated with a sense of achievement in females under 50 years old (Figure 2 and Figure 3, bottom left). In contrast, no item is associated with psychosomatic disorders, and three items (canned tuna (amount), canned tuna (freq), and squid (amount)) are negatively correlated with a sense of achievement in males under 50 years old (Figure 3, top left).

For fruits, the frequencies of melon and watermelon consumption are positively correlated with a sense of achievement in females under 50 years old (Figure 3, bottom left). The amounts of pineapple and watermelon consumed per occasion are negatively correlated with psychosomatic disorders in males under 50 years old and those aged 50 and older, respectively (Figure 2, top). The frequency of strawberry consumption is negatively correlated with a sense of achievement in females aged 50 and older (Figure 3, bottom right).

For vegetables and Japanese pickles, many food items are negatively correlated with psychosomatic disorders in males, irrespective of age group (Figure 2, top). In addition, some food items are positively correlated with a sense of achievement in females, again irrespective of age group (Figure 3, bottom).

For cereals, several food items are positively associated with psychosomatic disorders in individuals, except for females under 50 years old, and are positively associated with a sense of achievement in females. For example, the amount of bread consumed per occasion is positively correlated with psychosomatic disorders in males, irrespective of age group (Figure 2, top). The amount of Japanese noodles (Udon) consumed per occasion is positively correlated with psychosomatic disorders in males (Figure 2, top right) and with a sense of achievement in females (Figure 3, bottom right), both for individuals aged 50 and older.

Potatoes show an association with our regressands only in males under 50 years old: the amounts of sweet potatoes and taros consumed per occasion are negatively correlated with a sense of achievement in them (Figure 3, top left).

For soybean products, the frequency of boiled tofu and the amount of fried bean curd consumed per occasion are negatively correlated with psychosomatic disorders in males under 50 years old (Figure 2, top left). The frequency of tofu in miso soup is negatively correlated with a sense of achievement in males under 50 years old (Figure 3, top left). The frequency and the per occasion amount of fermented soybeans show different directions of correlation with a sense of achievement in females under 50 years old: the former is negative, and the latter is positive (Figure 3, bottom left).

For confectioneries, some food items are positively correlated with psychosomatic disorders in males aged 50 and older, and negatively correlated with them in both males and females under 50 years old. For example, the amount of Japanese cake and rice crackers consumed per occasion and the frequency of snack consumption are positively correlated with psychosomatic disorders in males aged 50 and older (Figure 2, top right). The frequency of chocolate consumption in males under 50 years old and the frequency of snack consumption and the amount of rice crackers consumed per occasion in females under 50 years old are negatively correlated with psychosomatic disorders (Figure 2, left).

With regard to miscellaneous items, margarine (frequency and amount), mayonnaise (amount), ketchup (frequency), mustard (frequency), and wasabi (amount) are negatively associated with psychosomatic disorders or positively associated with a sense of achievement in males, regardless of age group, but not in females (Figure 2 and Figure 3).

Finaly, we also conducted a power analysis for our logistic regression. The power for several setups is shown in Supplementary Table S2. When *n* = 50, the power of a 95% significance test for odds is 58.6% with odds of 0.6, or 30.4% with odds of 1.4. When *n* = 200, the power of a 95% significance test for odds is 99.9% with odds of 0.6, or 88.5% with odds of 1.4. These results imply that the type II error is not small for males. Interestingly, according to Figure 2 and Figure 3, we observed many more significant odds in the smaller subsample (i.e., males) compared to the larger subsample (i.e., females). The results of the power analysis reinforce the regression findings, indicating that many more food items are associated with physical and mental disorders or a sense of achievement for males than for females.

## 4. Discussion

Our results relating to vegetable intake are consistent with previous research [31]. Many vegetable items are positively associated with a sense of achievement and negatively associated with psychosomatic disorders. The positive correlation between mental health and whole foods, such as fruits, vegetables, and whole grains [19,20], was confirmed in the male subsample, but the results were weaker in the female subsample.

Although dietary fish intake has been reported to positively associate with work engagement for Japanese workers [32], our results indicate that many of fish and seafood items are negatively associated with a sense of achievement and positively associated with psychosomatic disorders. Our sample includes only 267 (37.1%) full-time workers, and locationally restricted to a city, Ebetsu, Hokkaido.

For some FFQ items, the correlation with psychosomatic disorders or a sense of accomplishment varies across different subsamples. For example, the correlation between the frequency of onion consumption and a sense of accomplishment is negative for males aged 50 and older but positive for females under 50 years old. Such food items include snacks (freq), rice crackers (amount), oolong (ready-made), and tap water for psychosomatic disorders, and fried chicken (freq) and eating out (freq) for a sense of achievement. A possible explanation for these results is based on the fact that the relationship between food and mental condition is bidirectional. The results will differ in sign for each subsample depending on the relative magnitudes of the two directional relationships.

For some food items, data on both the frequency of consumption occasions and the amount consumed per occasion are gleaned from the FFQ. Thus, the variation in food consumption patterns, even within groups consuming the same total amount of food per period, can be captured by the FFQ. In our logit regression analysis results, both aspects show either the same or different directions of correlation with the regressands within the subsamples for several food items.

The frequency and per occasion amount of fermented soybeans show different directions of correlation with a sense of achievement in females under 50 years old: the former is negative, and the latter is positive. In Japan, it is common for fermented soybeans to be sold in individual servings at the retail level. About 80% of individuals in our sample reported eating the standard amount of fermented soybeans per occasion. The amount of fermented soybeans consumed per occasion might serve as a proxy variable for purchasing behavior, particularly persistence with minor suppliers who sell the product in larger portions. Of course, there is a possibility that some individuals eat two or more packages of fermented soybeans per occasion.

The frequency and per occasion amount show the same direction of correlation with our regressands for seven food items: pork liver (positive with a sense of achievement in both males and females aged 50 and older), grilled chicken (positive with psychosomatic disorders in males aged 50 and older), fried chicken (positive with psychosomatic disorders in males aged 50 and older), milk (positive with psychosomatic disorders in females under 50 years old), canned tuna (negative with a sense of achievement in males under 50 years old), horse mackerel and sardine (positive with psychosomatic disorders in females under 50 years old), and spinach (negative with psychosomatic disorders in males aged 50 and older). This pattern implies that the total amounts of consumption of these food items per period are associated with psychosomatic disorders or a sense of achievement,. rather than the distribution of consumption occasions and amounts per occasion

The limitations of our research are as follows: First, we conducted exploratory analysis using logistic regressions for each FFQ item. Although we controlled for some confounders in every regression, there remains a risk of omitted variable bias. For example, FFQ items include both roasted coffee beans and ready-made coffee. These two items might serve as proxies for the cultural background of respondents’ households.

Second, our dummy variable regressands entail the loss of information. These regressands are the indicators of whether the individual scale of psychosomatic disorders or the sense of achievement is above or below the median of the subsample. Original measures for psychosomatic disorders or a sense of achievement include the eleven-point Likert scale.

Third, our sample for the analysis is limited to a specific area in Japan. Although the food supply system is ubiquitous throughout Japan, local traditional dietary habits cannot be ignored.

## 5. Conclusions

We explored the relationship between dietary habits and psychosomatic disorders or a sense of achievement using the survey data for males and females in Ebetsu city and its surrounding area, Hokkaido. Especially, we considered the dietary habits in terms of the amount per occasion and the frequency of intake at the foodstuff level. Compared to existing studies, the unique aspect of this research is the vast number of candidate explanatory variables (FFQ items). In addition to confirming several findings from leading research, our results include new aspects of the relationship between food intake and physical or mental condition: (i) the manner of food intake, i.e., the frequency and amount per occasion in our analysis, can influence the association between food and stress condition; (ii) the bidirectional interaction between food and stress condition creates distinct characteristics in the relationship between food and stress for each subpopulation.

## Figures and Tables

**Table 1:** Summary statistics.

	**Male**								
	**Age < 50**				**Age ≥ 50**				
	**(*n* = 68)**				**(*n* = 94)**				
	**Mean**	**Std.**	**Min**	**Max**	**Mean**	**Std.**	**Min**	**Max**	
Age	41.10	6.93	21	49	59.90	6.67	50	75	
MBI	22.96	4.34	16.2	43.8	24.08	3.13	17.2	36.1	*p* < 0.1
SBP	120.63	15.95	95	176	133.22	18.69	94	190	*p* < 0.0001
House mate (spouse)	0.79	0.41	0	1	0.86	0.35	0	1	
House mate (child)	0.49	0.50	0	1	0.38	0.49	0	1	
House mate (parent)	0.13	0.34	0	1	0.07	0.26	0	1	
School (junior college)	0.19	0.40	0	1	0.07	0.26	0	1	*p* < 0.05
School (university)	0.66	0.48	0	1	0.72	0.45	0	1	
Full-time worker	0.74	0.44	0	1	0.56	0.50	0	1	*p* < 0.05
Psychosomatic	3.40	2.16	0	10	3.05	2.77	0	10	
Achievement	5.72	2.04	0	9	6.34	2.39	0	10	*p* < 0.1
Y1≡1 [Psychosomatic ≥ median]	0.60	0.49	0	1	0.66	0.48	0	1	
Y2≡1 [Achievement < median]	0.41	0.50	0	1	0.44	0.50	0	1	
	**Female**								
	**Age < 50**				**Age ≥ 50**				
	**(*n* = 222)**				**(*n* = 270)**				
	**Mean**	**Std.**	**Min**	**Max**	**Mean**	**Std.**	**Min**	**Max**	
Age	40.69	6.98	21	49	57.78	5.98	50	76	
MBI	21.13	3.52	15.3	38.6	21.43	3.01	15	31.7	
SBP	107.68	13.61	81	161	119.32	16.88	80	199	*p* < 0.0001
House mate (spouse)	0.53	0.50	0	1	0.66	0.48	0	1	*p* < 0.01
House mate (child)	0.47	0.50	0	1	0.43	0.50	0	1	
House mate (parent)	0.19	0.39	0	1	0.15	0.36	0	1	
School (junior college)	0.48	0.50	0	1	0.51	0.50	0	1	
School (university)	0.36	0.48	0	1	0.19	0.39	0	1	*p* < 0.0001
Full-time worker	0.38	0.49	0	1	0.18	0.38	0	1	*p* < 0.0001
Psychosomatic	4.48	2.81	0	10	4.00	2.74	0	10	*p* < 0.1
Achievement	5.82	2.15	0	10	5.93	2.15	0	10	*p* < 0.1
Y1≡1 [Psychosomatic ≥ median]	0.57	0.50	0	1	0.64	0.48	0	1	
Y2≡1 [Achievement < median]	0.45	0.50	0	1	0.43	0.50	0	1	

Notes: House mate (spouse/child/parent) = an indicator of a spouse, child, or parent as a house mate; School (junior college/university) = an indicator of junior college or university education; Full-time worker = an indicator of full-time employment; Psychosomatic = an eleven-point Likert scale for psychosomatic disorders; Achievement = an eleven-point Likert scale for a sense of achievement. The right-most column shows *p*-values of the mean comparison *t*-test.

## Data Availability

The data obtained from the “*Sukoyaka* Health Survey” are available in a publicly accessible repository managed by the DNA Data Bank of Japan (DDBJ) Japanese Genotype-phenotype Archive at https://www.ddbj.nig.ac.jp/jga/index-e.html, accessed on 12 July 2024.

## Supplementary Materials

The following supporting information can be downloaded at https://www.mdpi.com/article/10.3390/nu16213702/s1: Table S1: FFQ items; Table S2: Power analysis for logit regression; Table S3. Validation analysis for psychosomatic disorders and a sense of achievement; Figure S1: Odds of FFQ items on psychosomatic disorders; Figure S2: Odds of FFQ items on a sense of achievement. Reference [33] is cited in the supplementary materials.
