# Supplementary material for: Exploring the Relationship Between Dietary Habits and Perceptions of Mental and Physical Disorders, or a Sense of Accomplishment in Japan
Source: Nutrients. 2024 Oct 30;16(21):3702. doi: 10.3390/nu16213702 (PMC11548033; doi:10.3390/nu16213702)
Supplement: Supplementary file 1 [file nutrients-16-03702-s001.zip › nutrients-3253709-supplementary.pdf]

Supplementary

Table S1. FFQ items

(I) Alcohol	
1	alcohol drinking (freq)
2	rice wine
3	distilled spirit
4	beer
5	whiskey
6	wine
7	size of rice bowl
8	rice (amount)
9	miso soup (freq)
10	miso soup (amount)
11	salt content of miso soup
(II) Meats	
12	beefsteak(freq)
13	beefsteak(amount)
14	beef grilled(freq)
15	beef grilled(amount)
16	beef saute(freq)
17	beef saute(amount)
18	beef stewed(freq)
19	beef stewed(amount)
20	pork saute(freq)
21	pork saute(amount)
22	pork fried(freq)
23	pork fried(amount)
24	pork stewed(freq)
25	pork stewed(amount)
26	pork simmered(freq)
27	pork simmered(amount)
28	pork soup(freq)
29	pork soup(amount)
30	pork liver(freq)
31	pork liver(amount)
32	chicken grilled(freq)
33	chicken grilled(amount)
34	chicken saute(freq)
35	chicken saute(amount)
36	chicken simmered(freq)
37	chicken simmered(amount)
38	chicken fried(freq)
39	chicken fried(amount)
40	chicken liver(freq)
41	chicken liver(amount)
42	ham (freq)
43	ham (amount)
44	sausage (freq)
45	sausage (amount)
46	bacon (freq)
47	bacon (amount)
(III) Dairy products and eggs	
48	low fat milk(freq)
49	low fat milk(amount)
50	milk(freq)
51	milk(amount)
52	egg (freq)
53	egg (amount)
54	cheeses (freq)
55	cheeses (amount)
56	yogurt (freq)
57	yogurt (amount)
(IV) Seafood	
58	salted fish (freq)
59	salted fish (amount)
60	dried fish (freq)
61	dried fish (amount)
62	canned tuna (freq)
63	canned tuna (amount)
64	salmon, trout (freq)
65	salmon, trout (amount)
66	tunas, bonito (freq)
67	tunas, bonito (amount)
68	amberjack (freq)
69	amberjack (amount)
70	pollack, flatfish (freq)
71	pollack, flatfish (amount)
72	sea breams (freq)
73	sea breams (amount)
74	horse mackerel, sardine (freq)
75	horse mackerel, sardine (amount)
76	pacific saury, mackerel (freq)
77	pacific saury, mackerel (amount)
78	shirasuboshi (freq)
79	shirasuboshi (amount)
80	cod roe, salmon roe (freq)
81	cod roe, salmon roe (amount)
82	eel (freq)
83	eel (amount)
84	squid (freq)
85	squid (amount)
86	octopus (freq)
87	octopus (amount)
88	shrimp (freq)
89	shrimp (amount)
90	clam, corb shell (freq)
91	clam, corb shell (amount)
92	fish sausage (chikuwa) (freq)
93	fish sausage (chikuwa) (amount)
94	boiled fish paste (kamaboko) (freq)
95	boiled fish paste (kamaboko) (amount)
96	fried fish paste (satsuma-age) (freq)
97	fried fish paste (satsuma-age) (amount)
(V) Fruits	
98	mandarin (freq)
99	mandarin (amount)
100	other oranges (freq)
101	other oranges (amount)
102	apple (freq)
103	apple (amount)
104	persimmon (freq)
105	persimmon (amount)
106	strawberry (freq)
107	strawberry (amount)
108	grapes (freq)
109	grapes (amount)
110	melon (freq)
111	melon (amount)
112	watermelon (freq)
113	watermelon (amount)
114	peach (freq)
115	peach (amount)
116	pears (freq)
117	pears (amount)
118	kiwi fruit (freq)
119	kiwi fruit (amount)
120	pineapple (freq)
121	pineapple (amount)
122	banana (freq)
123	banana (amount)
(VI) Pickles	
124	pickled radish(freq)
125	pickled radish(amount)
126	pickled green vegetables(freq)
127	pickled green vegetables(amount)
128	pickled plum(freq)
129	pickled plum(amount)
130	pickled Chinese cabbage(freq)
131	pickled Chinese cabbage(amount)
132	pickled cucumber(freq)
133	pickled cucumber(amount)
134	pickled eggplant(freq)
135	pickled eggplant(amount)
136	pickled turnip(freq)
137	pickled turnip(amount)
(VII) Vegetables	
138	carrot (freq)
139	carrot (amount)
140	spinach (freq)
141	spinach (amount)
142	pumpkin (freq)
143	pumpkin (amount)
144	cabbage (freq)
145	cabbage (amount)
146	radish (freq)
147	radish (amount)
148	green pepper (freq)
149	green pepper (amount)
150	tomatoes (freq)
151	tomatoes (amount)
152	long green onion (freq)
153	long green onion (amount)
154	leek (freq)
155	leek (amount)
156	green chive (freq)
157	green chive (amount)
158	green vegetable (shungiku) (freq)
159	green vegetable (shungiku) (amount)
160	green vegetable (komatsuna) (freq)
161	green vegetable (komatsuna) (amount)
162	broccoli (freq)
163	broccoli (amount)
164	onion (freq)
165	onion (amount)
166	cucumber (freq)
167	cucumber (amount)
168	eggplant (freq)
169	eggplant (amount)
170	Chinese cabbage (freq)
171	Chinese cabbage (amount)
172	burdock (freq)
173	burdock (amount)
174	bean sprout (freq)
175	bean sprout (amount)
176	snap bean (freq)
177	snap bean (amount)
178	lettuce (freq)
179	lettuce (amount)
180	green asparagus (freq)
181	green asparagus (amount)
182	garlic (freq)
183	garlic (amount)
(VIII) Cereals and confectionaries	
184	bread (freq)
185	bread (amount)
186	Japanese noodles (udon) (freq)
187	Japanese noodles (udon) (amount)
188	Japanese noodles (soba) (freq)
189	Japanese noodles (soba) (amount)
190	Chinese noodles (freq)
191	Chinese noodles (amount)
192	pasta (freq)
193	pasta (amount)
194	Japanese noodles (soumen) (freq)
195	Japanese noodles (soumen) (amount)
196	rice cake (freq)
197	rice cake (amount)
198	Japanese cake (freq)
199	Japanese cake (amount)
200	cakes (freq)
201	cakes (amount)
202	biscuit, cookie (freq)
203	biscuit, cookie (amount)
204	chocolate (freq)
205	chocolate (amount)
206	ice cream (freq)
207	ice cream (amount)
208	snacks (freq)
209	snacks (amount)
210	rice cracker (freq)
211	rice cracker (amount)
212	sesame (freq)
213	sesame (amount)
214	peanuts (freq)
215	peanuts (amount)
(IX) Soy bean products	
216	tofu in miso soup (freq)
217	tofu in miso soup (amount)
218	boiled tofu (freq)
219	boiled tofu (amount)
220	bean curd (koya-tofu) (freq)
221	bean curd (koya-tofu) (amount)
222	fried bean curd (freq)
223	fried bean curd (amount)
224	deep-fried bean curd (freq)
225	deep-fried bean curd (amount)
226	fermented soybeans (freq)
227	fermented soybeans (amount)
(X) Potatos	
228	sweet potatoes (freq)
229	sweet potatoes (amount)
230	potatoes (freq)
231	potatoes (amount)
232	taros (freq)
233	taros (amount)
234	yams (freq)
235	yams (amount)
236	konjac (freq)
237	konjac (amount)
(XI) Other foodstuffs	
238	mushroom (shitake) (freq)
239	mushroom (shitake) (amount)
240	mushroom (enokitake) (freq)
241	mushroom (enokitake) (amount)
242	mushroom (shimeji) (freq)
243	mushroom (shimeji) (amount)
244	seaweed (wakame) (freq)
245	seaweed (wakame) (amount)
246	seaweed (hijiki) (freq)
247	seaweed (hijiki) (amount)
248	dried seaweed (nori) (freq)
249	dried seaweed (nori) (amount)
250	butter (freq)
251	butter (amount)
252	margarine (freq)
253	margarine (amount)
254	jam (freq)
255	jam (amount)
256	salad dressings (freq)
257	salad dressings (amount)
258	mayonnaise (freq)
259	mayonnaise (amount)
260	Worcester sauce (freq)
261	Worcester sauce (amount)
262	ketchup (freq)
263	ketchup (amount)
264	mustard (freq)
265	mustard (amount)
266	wasabi (freq)
267	wasabi (amount)
(XII) Beverages	
268	Japanese tea (leaf)
269	Japanese tea (ready-made)
270	oolong (leaf)
271	oolong (ready-made)
272	red tea (leaf)
273	read tea (ready-made)
274	coffee (beans)
275	coffee (instant)
276	coffee (ready-made)
277	tomato juice
278	vegetable juice
279	100% orange juice
280	100% apple juice
281	100% grapefruits juice
282	other fruit juice
283	carbonated drink
284	soybean juice
285	latic drink
286	tap water
287	bottled water
288	sugar in tea
289	milk in tea
290	sugar in coffee
291	milk in coffee
(XIII) Miscellaneous FFQ items	
292	breakfast (freq)
293	eating-out (freq)
294	instant food (freq)
295	saue (freq)
296	fry (freq)
297	fatty meat (amount)
298	soup of noodle (amount)
299	table salt habit
300	table soy sauce habit
301	doneness of steak
302	baked fish (freq)
303	burned fish (amount)

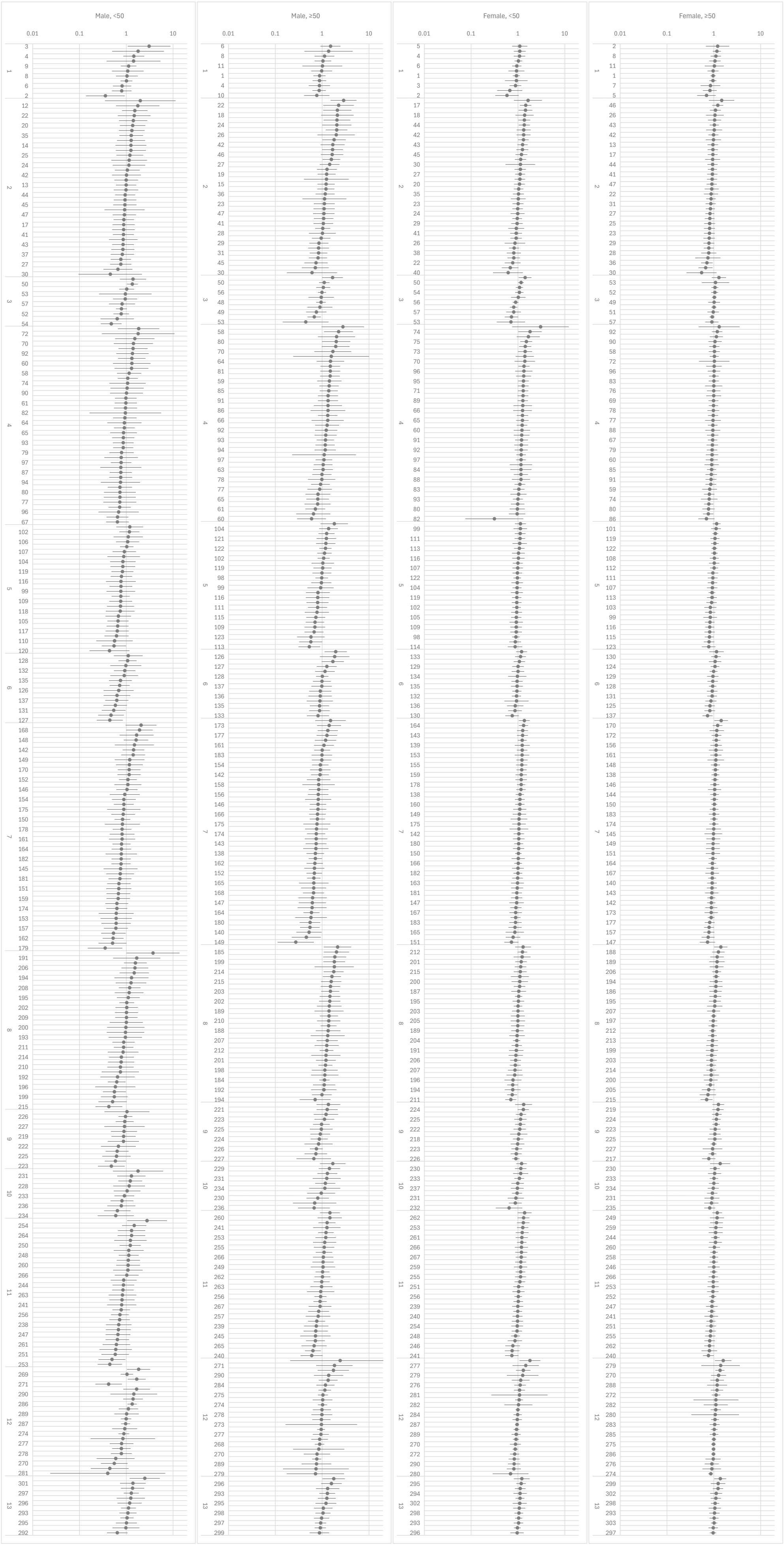

Figure S1. Odds of FFQ items on psychosomatic disorders

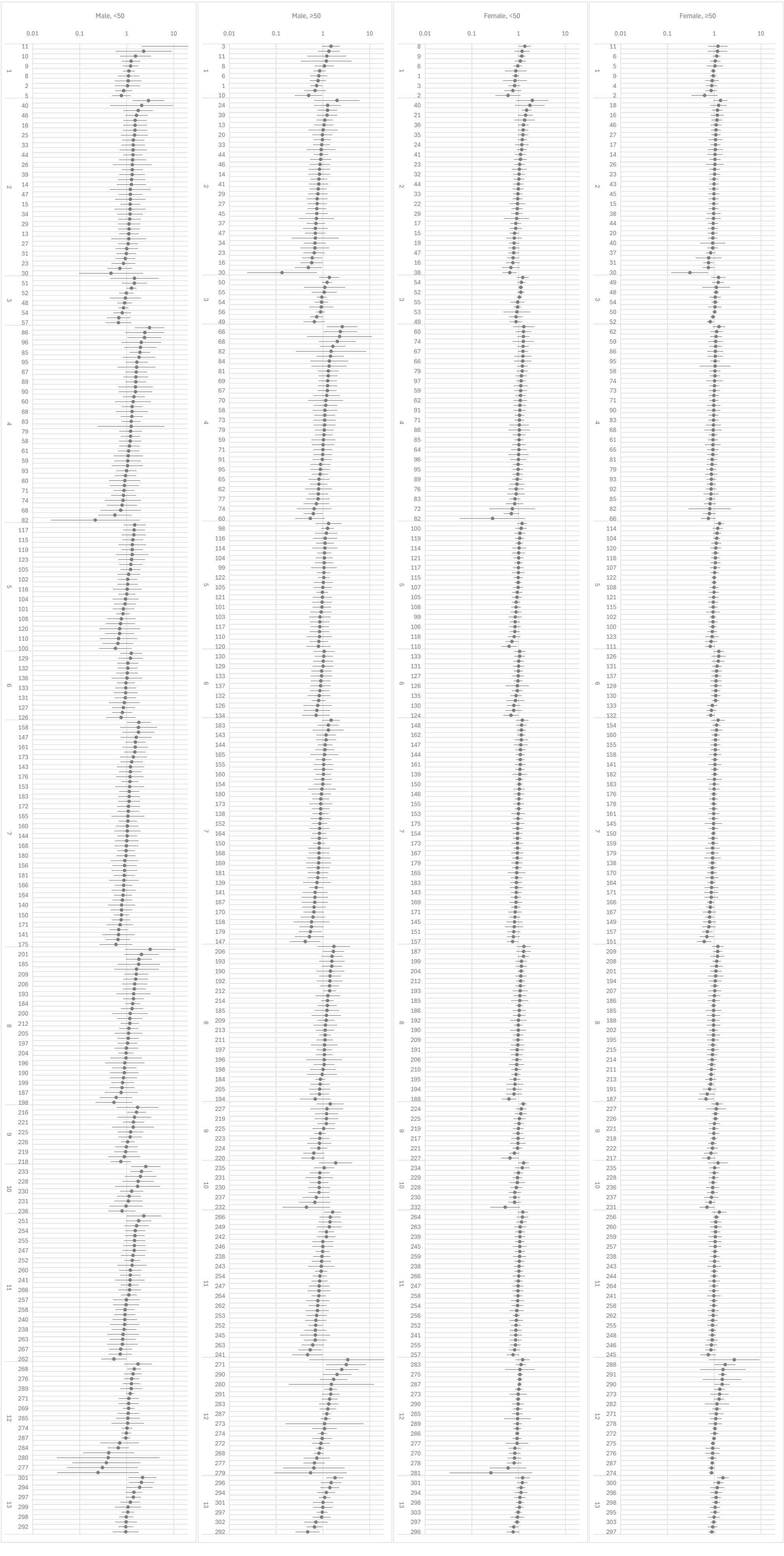

Figure S2. Odds of FFQ items on a sense of achievement

Table S2. Power analysis for logit regression

$\alpha = 0.05$	Odds=0.6	Odds=0.8	Odds=1.2	Odds=1.4
N=50	0.586	0.147	0.123	0.304
N=100	0.910	0.302	0.219	0.589
N=150	0.985	0.420	0.341	0.799
N=200	0.999	0.557	0.421	0.885
N=250	1.000	0.675	0.498	0.957

Note on the power analysis:

The data generating process is assumed that x_i follows a binomial distribution with n=9 and p=0.3 to mimic the empirical distribution of FFQ items. Suppose that the indicator variable y_i takes the value 1 with the probability

$$\Pr(y_i = 1|x_i) = \frac{\exp(-a + bx_i)}{1 + \exp(-a + bx_i)},$$

where b is effect size in our power analysis, and a is set so that the sample mean of y_i is the same value in our logit regression analysis (i.e., 0.5). We conducted a power analysis with significant level of $\alpha = 0.05$, effect sizes $b = [\ln(0.6), \ln(0.8), \ln(1.2), \ln(1.4)]$, and sample sizes $N = [50, 100, 150, 200, 250]$.

Table S3. Validation analysis for psychosomatic disorders and a sense of achievement

Psychosomatic disorders

	Male, <50		Male, ≥50		Female, <50		Female, ≥50	
	p1	p2	p1	p2	p1	p2	p1	p2
Summer								
Vigor	0.999	0.001	1.000	0.000	1.000	0.000	1.000	0.000
Irritability	0.009	0.991	0.000	1.000	0.000	1.000	0.000	1.000
Fatigue	0.001	0.999	0.000	1.000	0.000	1.000	0.000	1.000
Anxiety	0.002	0.998	0.000	1.000	0.000	1.000	0.000	1.000
Depression	0.005	0.995	0.000	1.000	0.000	1.000	0.000	1.000
Physical	0.000	1.000	0.000	1.000	0.000	1.000	0.000	1.000
Winter								
Vigor	1.000	0.000	1.000	0.000	1.000	0.000	1.000	0.000
Irritability	0.033	0.967	0.000	1.000	0.000	1.000	0.000	1.000
Fatigue	0.000	1.000	0.000	1.000	0.000	1.000	0.000	1.000
Anxiety	0.005	0.995	0.000	1.000	0.000	1.000	0.000	1.000
Depression	0.000	1.000	0.000	1.000	0.000	1.000	0.000	1.000
Physical	0.004	0.996	0.000	1.000	0.000	1.000	0.000	1.000

A sense of achievement

	Male, <50		Male, ≥50		Female, <50		Female, ≥50	
	p1	p2	p1	p2	p1	p2	p1	p2
Summer								
Vigor	0.001	0.999	0.000	1.000	0.000	1.000	0.000	1.000
Irritability	0.870	0.132	1.000	0.000	1.000	0.000	0.998	0.002
Fatigue	0.779	0.224	1.000	0.000	1.000	0.000	0.871	0.129
Anxiety	0.949	0.052	1.000	0.000	1.000	0.000	0.995	0.005
Depression	0.977	0.024	1.000	0.000	1.000	0.000	1.000	0.000
Physical	0.790	0.213	1.000	0.000	0.988	0.012	0.992	0.009
Winter								
Vigor	0.003	0.998	0.000	1.000	0.000	1.000	0.000	1.000
Irritability	0.990	0.010	0.824	0.178	0.876	0.125	1.000	0.000
Fatigue	0.961	0.040	1.000	0.000	1.000	0.000	0.995	0.005
Anxiety	0.986	0.015	0.999	0.001	1.000	0.000	1.000	0.000
Depression	0.999	0.001	1.000	0.000	1.000	0.000	1.000	0.000
Physical	0.997	0.003	0.976	0.024	0.998	0.002	0.995	0.005

Note on the validation analysis:

Table S3 shows the p-values of the Mann-Whitney U test for two samples divided by the indicator variables $Y_1 \equiv 1$[psychosomatic disorders ≥ median] or $Y_2 \equiv 1$[a sense of achievement < median]. In the table, p1 and p2

correspond to the null hypotheses H0: ∃u, F(u) ≤ G(u) and H0: ∃u, F(u) ≥ G(u), where F and G are the cumulative distribution functions of the variables in the row header conditional on $Y_1(Y_2) = 0$ for F and $Y_1(Y_2) = 1$ for G, respectively. The row variables are the outcome scores from the Brief Job Stress Questionnaire (BJSQ) (Inoue et al., 2014). The variables in the table are measured over the past month during both summer and winter, whereas the variables for psychosomatic disorders and a sense of achievement in our paper are measured over the past year. This is because the variables in the BJSQ are only measured for the past month, but during both summer and winter.

According to the results, the variable for psychosomatic disorders is strongly related to all BJSQ stress outcomes. The variable for a sense of achievement is strongly related to the BJSQ outcome of 'vigor' across all subsamples.

Inoue, A.; Kawakami, N.; Shimomitsu, T.; Tsutsumi, A.; Haratani, T.; Yoshikawa, T.; Shimazu, A.; Odagiri, Y. Development of a short questionnaire to measure an extended set of job demands, job resources, and positive health outcomes: the new brief job stress questionnaire. Ind Health. 2014;52(3):175-89. doi: 10.2486/indhealth.2013-0185. Epub 2014 Feb 4. PMID: 24492763; PMCID: PMC4209588.
